# Clinically relevant plasma proteome for adiposity depots: evidence from systematic mendelian randomization and colocalization analyses

**DOI:** 10.1186/s12933-024-02222-1

**Published:** 2024-04-13

**Authors:** Min Cao, Bin Cui

**Affiliations:** 1 Department of Endocrine and Metabolic Diseases, Shanghai Institute of Endocrine and Metabolic Diseases, Ruijin Hospital, Shanghai Jiao Tong University School of Medicine, Shanghai, China; 2 Shanghai National Clinical Research Center for Metabolic Diseases, Key Laboratory for Endocrine and Metabolic Diseases of the National Health Commission of the PR China, Shanghai Key Laboratory for Endocrine Tumor, State Key Laboratory of Medical Genomics, Ruijin Hospital, Shanghai Jiao Tong University School of Medicine, Shanghai, China

**Keywords:** Adiposity depots, Plasma proteome, Mendelian randomization, Colocalization, Risk factors, Drug targets

## Abstract

**Background:**

The accumulation of visceral and ectopic fat comprise a major cause of cardiometabolic diseases. However, novel drug targets for reducing unnecessary visceral and ectopic fat are still limited. Our study aims to provide a comprehensive investigation of the causal effects of the plasma proteome on visceral and ectopic fat using Mendelian randomization (MR) approach.

**Methods:**

We performed two-sample MR analyses based on five large genome-wide association study (GWAS) summary statistics of 2656 plasma proteins, to screen for causal associations of these proteins with traits of visceral and ectopic fat in over 30,000 participants of European ancestry, as well as to assess mediation effects by risk factors of outcomes. The colocalization analysis was conducted to examine whether the identified proteins and outcomes shared casual variants.

**Results:**

Genetically predicted levels of 14 circulating proteins were associated with visceral and ectopic fat (*P* < 4.99 × 10^− 5^, at a Bonferroni-corrected threshold). Colocalization analysis prioritized ten protein targets that showed effect on outcomes, including FST, SIRT2, DNAJB9, IL6R, CTSA, RGMB, PNLIPRP1, FLT4, PPY and IL6ST. MR analyses revealed seven risk factors for visceral and ectopic fat (*P* < 0.0024). Furthermore, the associations of CTSA, DNAJB9 and IGFBP1 with primary outcomes were mediated by HDL-C and SHBG. Sensitivity analyses showed little evidence of pleiotropy.

**Conclusions:**

Our study identified candidate proteins showing putative causal effects as potential therapeutic targets for visceral and ectopic fat accumulation and outlined causal pathways for further prevention of downstream cardiometabolic diseases.

**Supplementary Information:**

The online version contains supplementary material available at 10.1186/s12933-024-02222-1.

## Introduction

Obesity is the most prevalent chronic disease worldwide. It is estimated that more than 1 billion people will be living with obesity by 2030 [1]. The fat deposited in people with obesity can be classified into subcutaneous adipose tissue (SAT), visceral adipose tissue (VAT), ectopic fat (including epicardial, liver, pancreatic and skeletal muscle fat) and others, according to their patterns of distribution [2]. The accumulation of VAT and ectopic fat is a key driver of cardiometabolic risk [3–6]. Therefore, therapeutic interventions aimed at reducing unnecessary visceral and ectopic fat may benefit cardiometabolic health.

Plasma proteins play a central role in many biological processes and represent a major source of therapeutic targets for many indications [7, 8]. Observational evidence in search of predictive circulating proteins for risk of VAT and ectopic fat has been limited due to small sample sizes [9, 10]. Moreover, the causal relevance of associations from these observational studies remains largely undetermined. Mendelian randomization (MR) is an established genetic epidemiology method that uses human genetics to ascertain whether a given biomarker is implicated in disease etiology [11]. Due to the rapidly increasing number of genome-wide association studies (GWAS) for plasma proteins [12–16], it is possible to implement an MR analysis to assess the causal association between plasma proteins and disease outcomes based on GWAS summary statistics. Several MR studies investigated the causal association between proteins and obesity traits. Zaghlool et al. [17] performed a bidirectional MR analysis to evaluate the causal association between over 1000 plasma proteins and body mass index (BMI) in ∼ 4600 participants, and identified a bi-directional causal relationship of BMI with leptin receptor (LEPR), insulin like growth factor binding protein 1 (IGFBP1) and netrin domain containing 2 (WFIKKN2), a protein-to-BMI relationship for advanced glycosylation end-product specific receptor (AGER), dermatopontin (DPT) and cathepsin A (CTSA), and a BMI-to-protein relationship for another 21 proteins. In addition, an MR study based on the Chinese Kadoorie Biobank (CKB) cohort explored the causal relationships between BMI and proteins, showing that the genetically predicted BMI was associated with six proteins, such as Interleukin-6 (IL-6), Interleukin-18 (IL-18) and C-C motif chemokine ligand 3 (CCL3) [18]. Goudswaard et al. [19] also performed an MR analysis between BMI and plasma proteins, and they found that BMI was causally associated with eight proteins, such as LEP, fatty acid binding protein 4 (FABP4), C5 (complement C5) and sex hormone binding globulin (SHBG). Besides BMI, the associations between proteome and other obesity-related phenotypes were also detected. A recent MR study based on GWAS summary statistics of 23 body composition traits and 2656 plasma proteins revealed 430 putatively causal effects of 96 plasma proteins on 22 body composition traits, including follistatin (FST) and IGFBP1 on trunk fat-free mass, and R-spondin 3 (RSPO3) on WHR [20]. Moreover, Zheng et al. [21] estimated the effects of 1,002 proteins on 225 phenotypes (including BMI and body fat) using MR, and identified three proteins - regulator of microtubule dynamics 1 (RMDN1), lactase (LCT) and programed cell death 1 ligand 2 (PDCD1LG2), which were causally associated with BMI. However, the MR evidence for proteome on adiposity depots is still scarce.

**Fig. 1:**
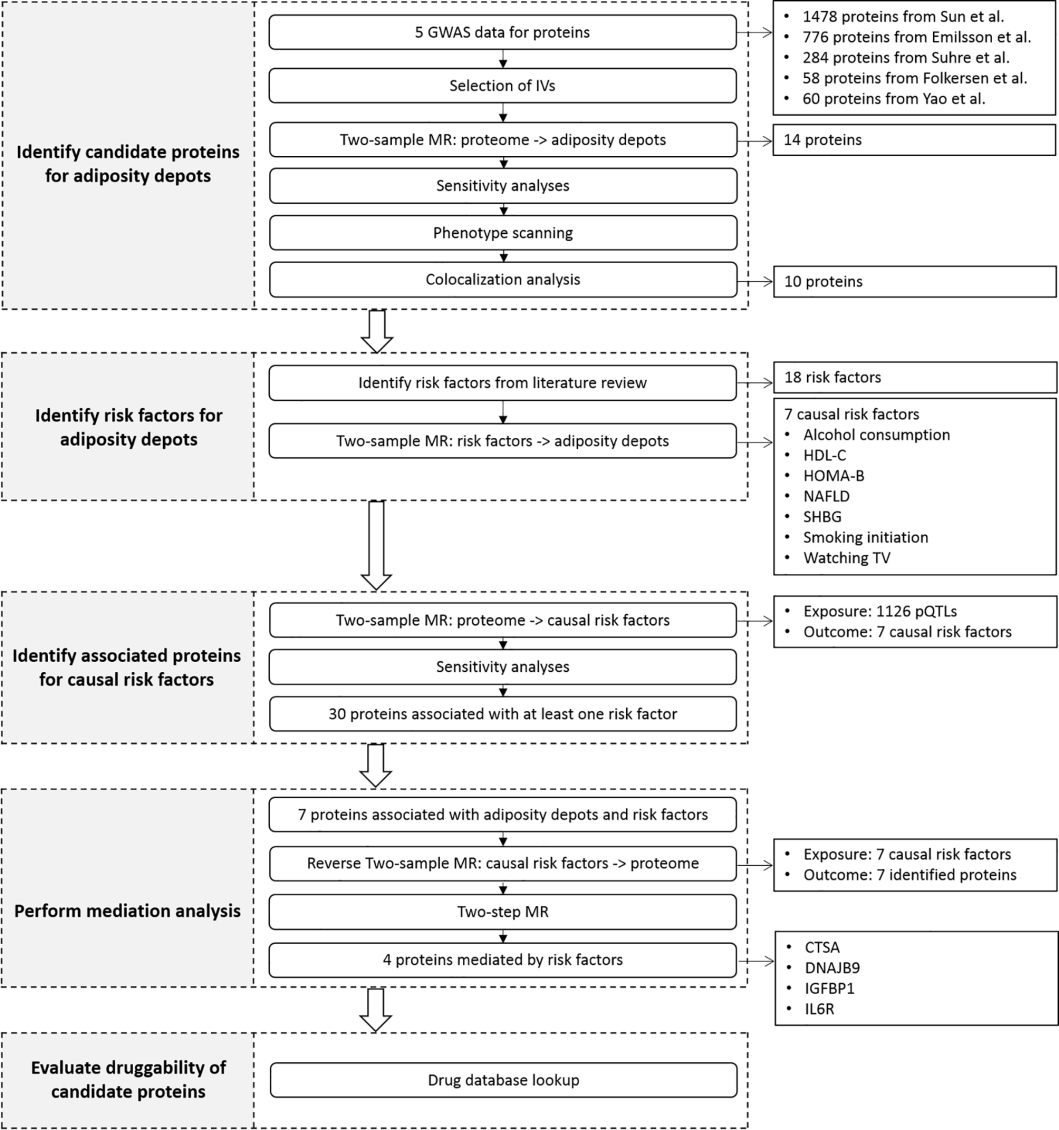
Overview of the study design. We identified circulating proteins and potential risk factors causally associated with visceral and ectopic fat using MR. We performed mediation analyses to estimate the risk factors mediating the effect of identified proteins on the outcomes using a two-step MR approach. We also screened the potentially targetable proteins through drug database searches

In this study, we systematically linked protein biomarkers to the outcomes of fat depots by taking a four-step approach. First, we applied a two-sample MR to estimate the causal effects of plasma proteins on visceral and ectopic fat, using genetic instruments (pQTLs) for up to 1002 proteins as exposure [12–16] and the summary statistics from a large European GWAS as the outcome [22]. Second, we performed colocalization analyses to verify the robustness of the instrumental variables. Third, we evaluated the causal relationship of proteins on the risk factors associated with visceral and ectopic fat and estimated the mediation effect. Last, we conducted phenotype scanning and characterized the druggability properties of these proteins as potential therapeutic targets for visceral and ectopic fat. Figure 1 summarizes the overall analysis pipeline applied in this study.

## Methods

### Proteomic data sources

MR instruments for circulating proteins were obtained from five proteomic GWAS [12–16]. All participants in the five studies were healthy Europeans. Circulating proteins in the GWAS by Sun et al. [12], Yao et al. [13], Suhre et al. [14] and Emilsson et al. [16] were measured using the SomaLogic platform, while in the GWAS by Folkersen et al. [15], the O-link platform was used. A total of 3606 pQTLs associated with 2656 plasma proteins have been identified.

For each plasma protein, pQTLs from its corresponding GWAS were used as genetic instruments. Consistent with Zheng et al. [21], IVs were selected using the following procedure: [1] SNPs associated with any protein (*P* ≤ 5 × 10^− 8^) in at least 1 of the 5 GWAS were included; [2] SNPs encoded by genes within the MHC region (chr6: from 26 Mb to 34 Mb) were excluded; [3] SNPs for each protein were clumped to retain independent SNPs only. The linkage disequilibrium threshold for clumping was set to r^2^ < 0.001; [4] SNPs associated with 5 or more proteins were excluded to avoid pleiotropy effect. Finally, a total of 1126 pQTLs were selected as potential IVs. We estimated the variance of each protein explained by its IVs by calculating the R^2^ [23] and the strength of each IV by the F-statistic [24]. IVs with an absolute F-statistic < 10 were considered as weak IVs and excluded from subsequent analyses. For pQTL that were not present in the outcome GWAS, we used proxy SNPs in high LD (R^2^ > 0.8) with the requested SNP. All palindromic SNPs as well as ambiguous SNPs were also excluded.

### Outcome data sources

To assess the association of pQTL with the risk of outcomes, we retrieved the effects of these pQTLs from a large GWAS in over 30,000 participants in the UK Biobank imaging cohort [21]. The estimates for the associations of IVs with outcomes were all of European ancestry will reduce the confounding by population stratification. The primary outcomes were visceral fat (visceral adipose tissue (VAT) volume, *N* = 32,860) and ectopic fat including: liver fat (*N* = 32,858), liver volume (*N* = 32,860), pancreas fat (*N* = 25,617) and pancreas volume (*N* = 31,758).

The secondary outcomes were the risk factors for visceral and ectopic fat, which were selected based on literature reviews [2, 25]. We sought well-powered publicly available GWAS summary statistics for these risk factors and removed risk factors that did not have GWAS data. The remaining 18 risk factors were considered for the two-sample MR analyses, including glycemic traits (homeostasis model assessment of insulin resistance [HOMA-IR] [26] and homeostasis model assessment of beta-cell function [HOMA-B] [26]), lipids [27] (high-density lipoprotein cholesterol [HDL-C], low-density lipoprotein cholesterol [LDL-C], total cholesterol [TC] and triglycerides [TG]), blood pressure [28](systolic blood pressure [SBP] and diastolic blood pressure [DBP]), nonalcoholic fatty liver disease [NAFLD] [29], sex hormones [30] (sex hormone binding globulin [SHBG] and total testosterone), lifestyle (moderate to vigorous physical activity [31], time spent watching television [30], coffee intake [30], smoking [32], and alcohol consumption [33]) and stress-related traits (insomnia [30] and major depression [34]). We used the same pQTLs as IVs for the secondary outcomes as for the primary outcomes.

### Systematic MR screening for causal proteins of outcome and risk factors

We performed two-sample MR analyses to estimate the effect of genetically predicted protein levels and on outcomes. The MR approach was based on the following assumptions: (i) the genetic variants used as an instrumental variable (IV) are associated with target exposure; (ii) there are no unmeasured confounders of the associations between genetic variants and outcome; (iii) the genetic variants are associated with the outcome only through changes in the exposure. We used the Wald’s ratio method to estimate the causal effects when there was only one IV available for target exposure, if more than one IV was available, we applied the inverse-variance weighting (IVW) method [35]. Additionally, various pleiotropy-robust MR methods (MR-Egger, weighted-median and weighted-mode) were performed in multi-instrument MR analyses [36]. We also performed two sensitivity analyses to evaluate the robustness of the identified causal associations for MR analyses: (i) MR-Egger regression analysis, which estimates an intercept term of the inverse variance weighted test, whose deviation from zero suggests the possibility of horizontal pleiotropy [36]; (ii) Cochran’s Q test, which calculates a statistic that measures heterogeneity across IVs [35]. 

We employed a two-sample MR framework incorporating the sensitivity analyses for both primary MR (proteins → outcomes of visceral & ectopic fat) and two-step MR (Step-1 MR: risk factors → outcomes of visceral & ectopic fat; Step-2 MR: proteins → risk factors). The MR methods applied in each of the MR settings depend on the number of IVs for each exposure. The results were presented as odds ratio (OR) with their 95% confidence intervals (CIs) scaled to a one standard deviation (SD) higher circulating protein level for the effects on binary outcomes, and presented as the effect size (95% CI) scaled to a 1-SD higher plasma protein levels for the effects on quantitative outcomes. Bonferroni correction was used to control for the total number of distinct proteins tested in our MR experiments (*P* < 4.99 × 10^− 5^ = 0.05/1002 proteins). The MR analyses were conducted using TwoSampleMR (version: 0.5.6) [37] package in R v4.0.3.

### Phenotype scanning

To further eliminate potential bias due to horizontal pleiotropy, we used PhenoScanner v2 [38] to assess any reported associations of the pQTL of the MR-prioritized proteins with potential confounders (*P* ≤ 1 × 10^− 8^), and with other circulating protein levels.

### Bayesian colocalization analysis

To assess potential confounding by LD, we checked whether the loci harboring the pQTL of the MR-prioritized proteins are also associated with outcomes of visceral & ectopic fat using colocalization as implemented in the coloc R package [39]. We conducted colocalization analysis for each of the 14 proteins associated with one or more of the outcomes to investigate whether the protein level and outcome genetic associations are due to the same causal variants. To estimate the posterior probability of each genomic locus containing a single variant affecting both the protein and outcomes, we analyzed all SNPs with minor allele frequency > 0.01 within 1 Mb of the pQTLs. In this study, we tested the posterior probability of hypothesis 3 (PPH3), in which both the protein and outcomes were associated with the region by different variants, and hypothesis 4 (PPH4), in which both the protein and outcomes were associated with the region by shared variants. We defined a gene as having evidence of colocalization based on gene-based PPH4 > 80% [40]. 

### Mediation analysis

For proteins that were causally associated with both visceral & ectopic fat outcomes and risk factors, we conducted a mediation analysis to quantify the effects of proteins on the outcomes via risk factors. Reverse MR between the mediator and identified proteins was conducted to determine whether there was bidirectionality that might affect the validity of the mediation model. The ‘total’ effect of exposure on outcome includes both ‘direct’ effect and any ‘indirect’ effect via one or more mediators. In this study, the total effect was captured by a standard univariable MR analysis - the primary MR. To decompose direct and indirect effects, we used results from two-step MR and chose the Product method to estimate the beta of indirect effect and the Delta method to estimate the standard error (SE) and confidence interval (CI) [41]. 

### Evaluation of druggability

To assess the druggability of identified proteins, we searched identified proteins in DrugBank and in a list of druggable genes from a previous study [42]. For proteins identified in drug database, information on drug molecule types, approved indications, and target outcomes in clinical trials was documented.

### Ethics approval

All the included studies had been approved by corresponding ethical review committees.

## Results

### Causal association between plasma proteins and outcomes of visceral and ectopic fat

**Fig. 2:**
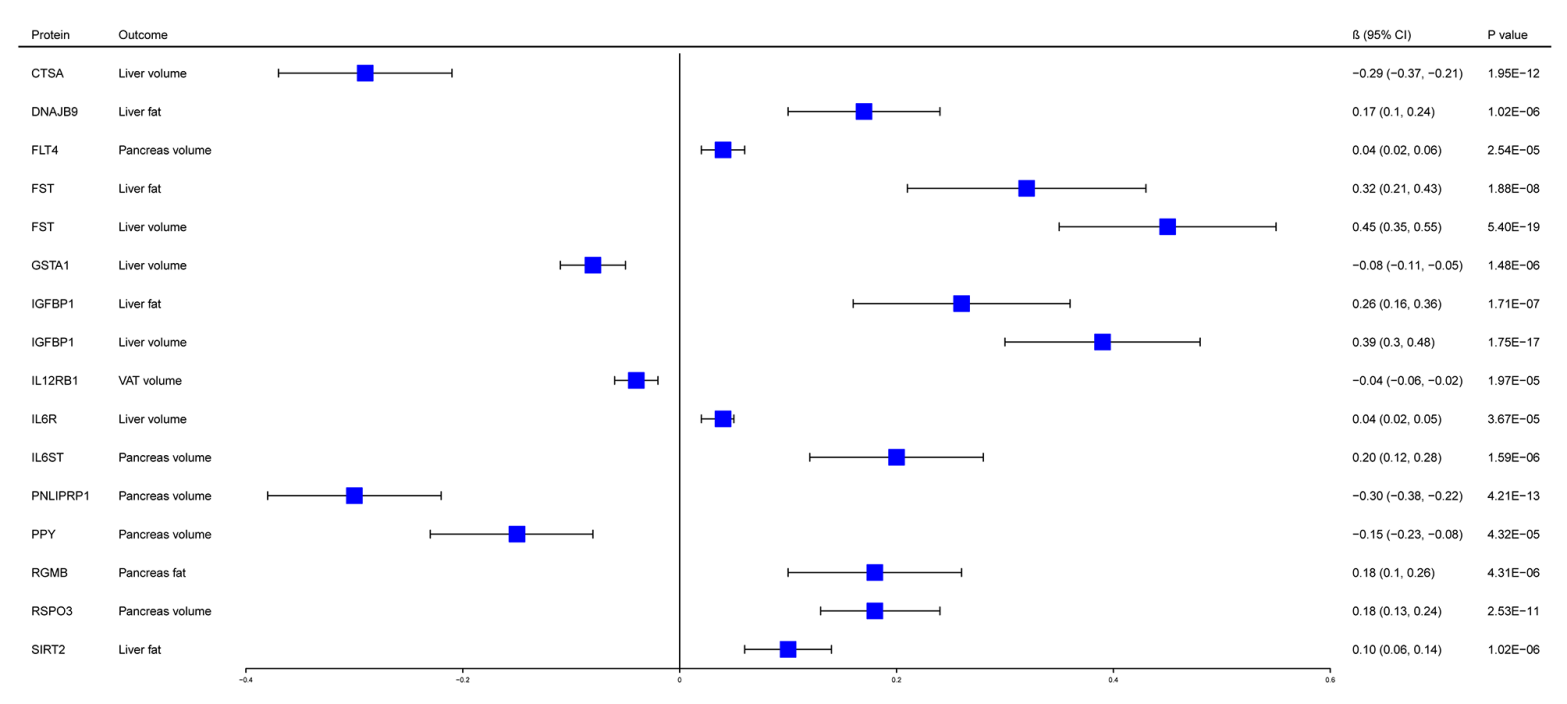
MR estimates of the causal associations of circulating proteins with outcomes of visceral and ectopic fat. MR estimates were derived from the IVW method. The data are presented as β coefficients (95% CIs), the squares represent β coefficients, and the error bars represent 95% CIs

We obtained 1126 pQTL from five proteomic GWAS and used them as instruments to evaluate the causal role of circulating proteins on outcomes. At the Bonferroni-corrected significance level (*P* < 4.99 × 10^− 5^), MR analyses revealed 14 putatively causal proteins with at least one outcome (Fig. 2, Supplementary Table 1), including: IL6R, GSTA1, RGMB, RSPO3, IL12RB1, CTSA, DNAJB9, FLT4, FST, IGFBP1, IL6ST, PNLIPRP1, PPY and SIRT2. Notably, IL12RB1 was associated with a lower risk of VAT volume (β[95% CI] = -0.04[-0.06, -0.02], *P* = 1.97 × 10^− 5^). Among the ectopic fat subtypes, FST, SIRT2, DNAJB9 and IGFBP1 were associated with a higher risk of liver fat; FST, IL6R and IGFBP1 were associated with a higher risk of liver volume, while CTSA and GSTA1 were associated with a lower risk of liver volume; RGMB was associated with a higher risk of pancreas fat (β[95% CI] = 0.18[0.10, 0.26], *P* = 4.31 × 10^− 6^); RSPO3, FLT4 and IL6ST were associated with a higher risk of pancreas volume, while PNLIPRP1 and PPY were associated with a lower risk of pancreas volume.

All pQTLs for the candidate proteins had an F-statistic > 10. Results of sensitivity analyses showed little evidence for heterogeneity based on Cochran Q statistics (Supplementary Table 1). The MR-Egger regression requires at least 3 IVs for analysis. Since we had at most 2 IVs for causal associations with strong MR evidence, we were not able to perform the MR-Egger analysis.

We also assessed whether the pQTL of proteins were associated with other phenotypes in PhenoScanner (Supplementary Table 2). We found that pQTL associated with GSTA1 (rs2290758) was not associated with any other traits, and was strongly associated with plasma GSTA1 levels, as well as two other proteins: GSTA2 and GSTA6P, suggesting potential pleiotropic effects. The pQTL for IL6R (rs4129267) was strongly associated with circulating IL6R, but was also associated with other proteins and traits. The pQTL for IL12RB1 (rs376008) was not associated with any other traits, but was associated with other proteins. Querying the pQTL for RGMB (rs1563317) in database, we noticed that it was strongly associated with RGMB levels and was not associated with any other traits or proteins, which reduces the probability that our MR estimate for RGMB was driven by horizontal pleiotropy. The pQTL for RSPO3 (rs2489623) was strongly associated with circulating IL6R and the other protein and trait (waist-to-hip ratio).

### Colocalization analysis

We conducted colocalization analyses of 14 causal proteins with visceral and ectopic fat. The high support of colocalization evidence was observed between three proteins (FST, SIRT2 and DNAJB9) and liver fat (PPH4 > 80%) (Supplementary Table 3), suggesting the associations in these regions were likely due to the same underlying causal variants. Similarly, FST, IL6R and CTSA were colocalized with liver volume, RGMB was colocalized with pancreas fat, and four proteins including PNLIPRP1, FLT4, PPY and IL6ST were colocalized with pancreas volume. The colocalization evidence at RSPO3, was less strong than with the other proteins, with colocalization PPH4 > 60% for pancreas volume, and there was no colocalization evidence for IGFBP1, GSTA1 and IL12RB1 with outcomes, implying a possible bias due to LD.

### Identification of likely causal risk factors for visceral and ectopic fat

To understand potential causal mechanisms between plasma proteins and outcomes of visceral and ectopic fat, we conducted two-step mediation MR analyses. First, we performed two-sample MR analyses to characterize the causal relationship of the potential risk factors with all the outcomes. Second, we assessed the causal effects of the proteins on the significant risk factors.

Of the 18 risk factors we considered, seven risk factors were causally associated with at least one of the outcomes (*P* < 0.05/18 = 0.0028, Bonferroni-adjusted for 18 risk factors; Table 1, Supplementary Table 4). The higher NAFLD risk was associated with a increased risk of liver fat (β[95% CI] = 0.40[0.33, 0.46]), while associated with a decreased risk of pancreas fat (β[95% CI] = -0.04[-0.06, -0.03]). Genetically predicted HDL-C was associated with a decreased risk of liver volume (β[95% CI] = -0.13[-0.21, -0.05]). Genetically predicted HOMA-B was associated with a decreased risk of liver fat (β[95% CI] = -0.06[-0.07, -0.04]). Genetically predicted SHBG was associated with a decreased risk of both liver fat (β[95% CI] = -0.21[-0.32, -0.09]) and liver volume (β[95% CI] = -0.14[-0.22, -0.06]). Moreover, the unhealthy lifestyle including longer time spent watching television, smoking initiation and alcohol consumption was associated with a higher risk of the outcomes. No association was observed between HOMA-IR, LDL, TC, TG, blood pressure, total testosterone, MVPA, coffee intake and stress-related traits (insomnia and major depression) with any of the outcomes.

**Table 1:** MR assessing the causal association between risk factors and primary outcomes

Exposure	Outcome	SNPs	IVW		Weighted median		Weighted mode		MR-Egger regression		Q	Q_ *P* value
			β (95% CI)	P	β	P	β	P	intercept	P		
alcohol consumption	Pancreas volume	3	0.359 (0.154, 0.564)	6.10e-04	0.395	5.05e-02	0.487	1.96e-01	0.007	0.845	0.671	7.15e-01
HDL-C	Liver volume	84	-0.129 (-0.212, -0.046)	2.37e-03	-0.069	3.21e-02	-0.065	3.45e-02	-0.004	0.216	384.565	2.39e-40
HOMA-B	Liver fat	3	-0.056 (-0.07, -0.043)	2.05e-15	-0.057	7.01e-01	-0.058	7.77e-01	-0.001	0.972	0.005	9.97e-01
NAFLD	Liver fat	2	0.395 (0.329, 0.461)	1.31e-31	…	…	…	…	…	…	6.666	9.83e-03
	Pancreas fat	2	-0.043 (-0.059, -0.028)	7.12e-08	…	…	…	…	…	…	0.309	5.78e-01
SHBG	Liver fat	150	-0.205 (-0.319, -0.091)	4.28e-04	-0.113	5.70e-03	-0.073	1.31e-01	-0.007	0.060	1161.315	2.79e-156
	Liver volume	150	-0.139 (-0.221, -0.057)	9.29e-04	-0.064	5.85e-02	-0.027	4.02e-01	-0.003	0.292	728.913	3.26e-77
smoking initiation	Liver volume	72	0.198 (0.11, 0.285)	9.56e-06	0.125	1.85e-02	0.096	3.32e-01	-0.007	0.208	111.529	1.52e-03
	Pancreas fat	72	0.137 (0.052, 0.222)	1.66e-03	0.129	4.05e-02	0.161	3.00e-01	-0.001	0.865	71.421	4.64e-01
	VAT volume	72	0.166 (0.087, 0.245)	3.78e-05	0.121	2.26e-02	0.053	5.99e-01	-0.002	0.770	101.869	9.57e-03
time spent watching television	Liver fat	52	0.407 (0.218, 0.596)	2.40e-05	0.345	1.15e-02	0.433	1.75e-01	-0.006	0.319	55.483	3.10e-01
	Pancreas fat	52	0.491 (0.251, 0.732)	6.26e-05	0.526	3.22e-04	0.632	3.84e-02	-0.008	0.310	73.890	1.97e-02
	VAT volume	52	0.656 (0.486, 0.826)	3.61e-14	0.632	1.22e-07	0.789	2.32e-02	-0.007	0.202	61.121	1.57e-01

### Identification of plasma proteins associated with risk factors for outcomes

We performed MR analyses of all 1126 pQTLs with the seven likely causal risk factors for the outcomes, and found 30 proteins were associated with at least one risk factor after multiple testing correction (*P* < 4.99 × 10^− 5^), of which 18 with HDL-C, 21 with SHBG, two with alcohol consumption, three with time spent watching television and five with smoking initiation (Supplementary Table 5). The instrument validity test presented sufficient instrument strength of all the pQTLs for MR analyses (F-statistic > 10), but sensitivity analyses showed potential heterogeneity for some of the tested associations.

Among the 14 proteins causally associated with outcomes of visceral and ectopic fat based on the primary MR analyses, seven proteins were found to be associated with one or more of the potential risk factors (Table 2). Of note, we found that genetically predicted higher CTSA was associated with higher HDL-C (β[95% CI] = 0.11 [0.07, 0.15], *P* = 1.16 × 10^− 8^) and SHBG (β[95% CI] = 0.51 [0.47, 0.54], *P* = 6.72 × 10^− 188^). Genetically determined higher DNAJB9 levels were associated with lower risk of SHBG (β[95% CI] = -0.12 [-0.15, -0.1], *P* = 2.09 × 10^− 21^). Genetically higher FST and IGFBP1 levels were both associated with lower risk of alcohol consumption and SHBG. Genetically higher IL12RB1 levels were associated with higher risk of SHBG. Genetically higher IL6R levels were associated with higher risk of SHBG. Genetically higher RSPO3 levels were associated with lower risk of HDL-C. The effect direction of the association of IL6R with SHBG was consistent with its association with liver volume, indicating that SHBG might be the potential mediator of the association between IL6R and liver volume (Table 2, Supplementary Table 1). In bidirectional MR analyses, there was little evidence that mediators decreased or increased these proteins significantly, except the associations between HOMA-B and CTSA, SHBG and FST, as well as NAFLD and RSPO3 (Supplementary Table 6).

**Table 2:** MR assessing the causal association between potential proteins and causal risk factors for outcomes

Protein	Outcome	β (95% CI)	SE	*P* value
CTSA	HDL-C	0.11 (0.07, 0.15)	0.020	1.16e-08
	SHBG	0.51 (0.47, 0.54)	0.017	6.72e-188
DNAJB9	SHBG	-0.12 (-0.15, -0.1)	0.013	2.09e-21
FST	alcohol consumption	-0.2 (-0.24, -0.16)	0.021	1.31e-21
	SHBG	-0.46 (-0.5, -0.41)	0.022	3.14e-99
IGFBP1	alcohol consumption	-0.17 (-0.21, -0.13)	0.019	3.31e-19
	SHBG	-0.4 (-0.44, -0.36)	0.019	1.20e-95
IL12RB1	SHBG	0.02 (0.02, 0.03)	0.004	4.17e-09
IL6R	SHBG	0.02 (0.01, 0.03)	0.004	4.49e-08
RSPO3	HDL-C	-0.07 (-0.1, -0.05)	0.013	5.76e-09

Among the 30 proteins that were associated with at least one stroke risk factor, 23 were found to be associated with the risk factors but not the primary outcomes of visceral and ectopic fat (Supplementary Table 5).

### Mediation effect of plasma proteins on outcomes via risk factors

**Fig. 3:**
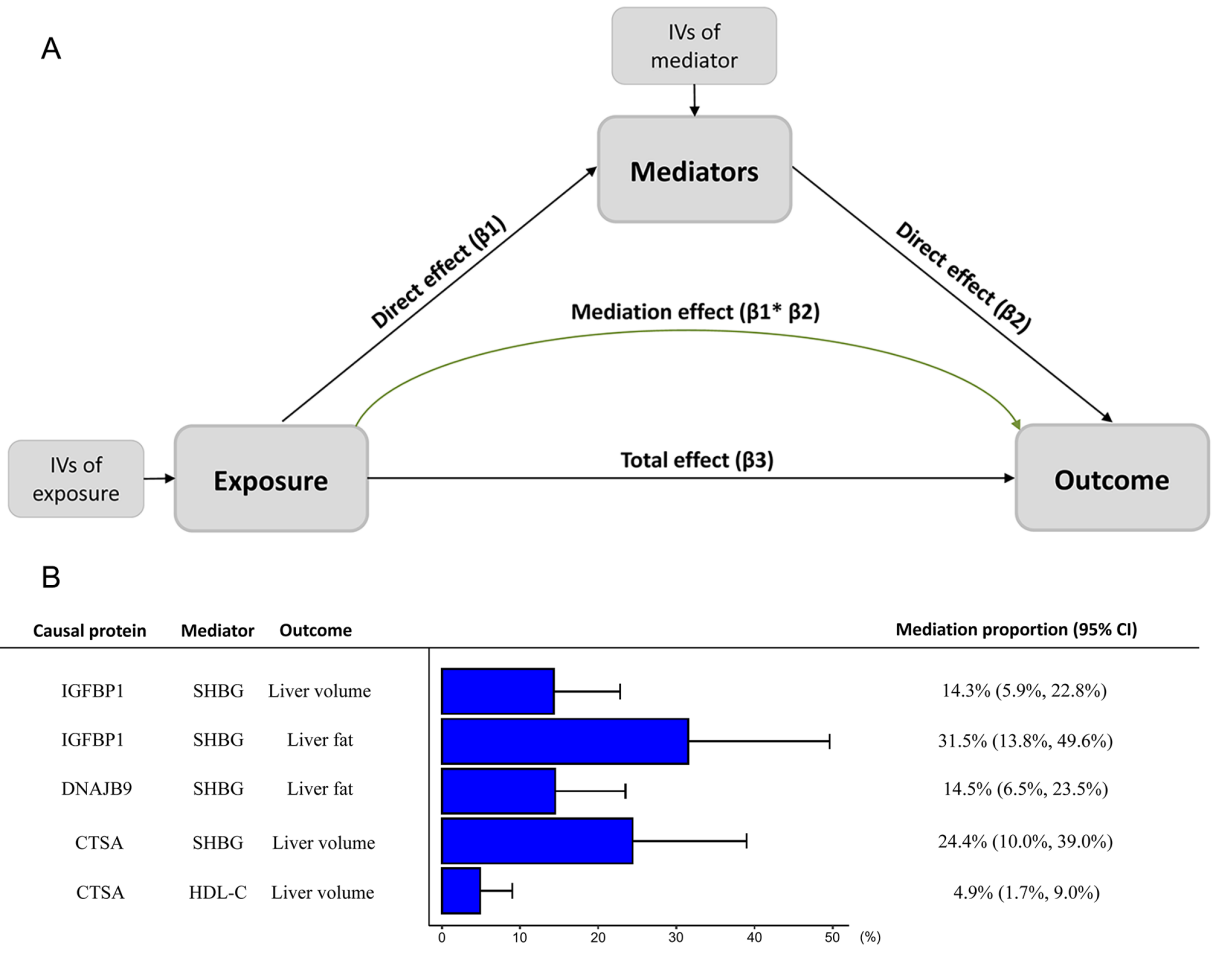
Mediating role of risk factors in the causal association between identified proteins and outcomes. **A)**, Two-step MR analysis framework. β1 effect of exposure on mediator, β2 effect of mediator on outcome, β3 effect of exposure on outcome. **B)**, MR estimates for the mediation effect of each mediator and the proportion mediated by each mediator. The squares and bars represent the mediated proportions (95% CIs)

Two-step MR analyses were conducted to estimate the mediation effect of each risk factor in the association between proteins and outcomes of visceral and ectopic fat. The proportion of mediation effect of DNAJB9 on liver fat via SHBG was 14.5%. The causal mediators from CTSA to liver volume was SHBG (24.4%) and HDL-C (4.9%) respectively. The proportion of mediation effect of IGFBP1 on liver fat via SHBG is 31.5%, and its effect on liver volume via SHBG was 14.3% (Fig. 3 and Supplementary Table 7).

### Druggability of identified proteins

We searched 14 circulating proteins identified through MR analysis as possible drug targets in drug database, and no direct evidence showed them as the drug targets for visceral fat or ectopic fat (Supplementary Table 8). However, the drug Mecasermin targeting IGFBP1 has been approved to treat growth failure in pediatric patients with primary IGF-1 deficiency or with growth hormone gene deletion and is likely to be used for treating obesity. Moreover, one drug targeting GSTA1 named Curcumin has been approved to treat type 2 diabetes. Drugs targeting SIRT2 and FLT4 were mainly designed to treat cancers. Two drugs targeting IL6R have been approved to treat rheumatoid arthritis. Several drugs targeting CTSA have been approved for the treatment of chronic hepatitis B virus infection, hepatitis C virus infection and RNA virus infections including COVID-19. No information was available for DNAJB9, RGMB, PNLIPRP1, RSPO3, PPY, IL6ST and IL12RB1 in the drug database.

## Discussion

We conducted two-sample MR and colocalization analyses to explore the causal roles of 1002 circulating proteins in visceral and ectopic fat to provide preclinical clues for the drug development. MR analysis identified 14 proteins causally associated with primary outcomes, and ten of them showed strong colocalization evidence. We found that seven risk factors includingHDL-C, HOMA-B, NAFLD, SHBG, alcohol consumption, smoking and time spent watching television were causally associated with primary outcomes, demonstrating a key role of these risk factors in the development of visceral and ectopic fat accumulation. We showed the associations of CTSA, DNAJB9 and IGFBP1 with primary outcomes were likely to be mediated by SHBG and HDL-C. Finally, we searched drug database for the selected proteins that may represent future intervention and treatment targets.

In the current study, we identified IL6R associated with increased risk of liver volume with robust evidence. Interleukin-6 (IL6), a proinflammatory cytokine, is shown to have strong associations with obesity, insulin resistance [43], type 2 diabetes [44] and liver disease [45, 46]. Several studies point to a role of IL-6 in the regulation of energy homeostasis and body weight through the classical IL6-IL6R signaling pathway [47–51]. Enhanced inflammation in liver and skeletal muscles and insulin resistance was observed in hepatocyte-specific IL6R deficient animals [52, 53]. Notably, a MR study reported inhibiting the IL6 signaling pathway via IL6R blockade might increase the risk of NAFLD, suggesting IL6R should play a protective role in NAFLD [54]. The above evidence supports the potential effect of IL6R on the risk of ectopic fat and is worthy of further studies.

In humans, circulating IGF1 is mostly produced by hepatocytes and over 99% bound to six different insulin growth factor-binding proteins (IGFBPs) [55]. IGFBP1 was involved in glucose metabolism [56] and molecular regulation of obesity [57]. A global IGFBP1 deletion in mice showed a significant increase in body weight and body fat mass [58]. In addition, IGFBP1 was identified as potential target in the previous MR analyses of plasma proteome in obesity [17, 20]. NAFLD patients with advanced fibrosis had higher levels of IGFBP1 [59]. In alcoholic cirrhosis, serum IGFBP1 levels was elevated when compared with controls [60, 61]. With our finding that IGFBP1 is associated with liver fat and volume, further investigation of IGFBP1 on ectopic fat is justified.

Follistatin (FST) was originally discovered as an endogenous inhibitor of follicle-stimulating hormone [62]. Liver is a key contributor to the circulating FST in humans [63]. FST can promote the differentiation and browning of adipocytes and mitigate systemic metabolic inflammation [64, 65]. Clinical studies showed that NAFLD patients had elevated circulating levels of FST [66]. The previous evidence supported that FST is associated with insulin resistance and energy metabolism [67], which is consistent with our results of the significant associations between FST and liver fat/volume, suggesting that FST might represents a potentially promising therapeutic target for ectopic fat.

Lysosomal protective protein (CTSA) is a lysosomal serine carboxypeptidase [68]. A recent MR study revealed that CTSA was potentially causal for the development of obesity [17]. Repulsive Guidance Molecule b (RGMB) is a member of the RGM family, which consists of RGMA, RGMB, and RGMC [69]. While RGMB neural expression in adult mice is limited to the basal ganglia and pituitary, the protein is expressed more broadly in the gut, bone, heart, lung, liver, kidney, testis, ovary, uterus, epididymis and pancreas of adult mice [70, 71], and humans [72, 73]. RGMB deficiency significantly altered the diversity of gut microbiota and also induced dysbiosis [74]. However, literature on the associations of both CTSA and RGMB with ectopic fat was scarce. Further cohort and functional studies are warranted.

Another noteworthy finding of this study is the identification and quantification of the mediating roles of risk factors in the association between proteome and adiposity depots. In the current study, we selected 18 candidate mediators covering lifestyle, metabolic and stress related traits, SHBG and HDL-C were finally identified as the causal mediators. Interestingly, SHBG plays a significant role in mediating the pathways between proteins and adiposity depots, with the proportion of mediation effect of IGFBP1 on liver fat 31.5%, DNAJB9 on liver fat 14.5%, IGFBP1 on liver volume 14.3%, and CTSA on liver volume 24.4%. These results are consistent with previous epidemiological and MR evidence that SHBG was associated with lower liver fat [75–77], implicating the protective role of SHBG against liver fat accumulation. Cai et al. found that genetically determined circulating SHBG were inversely associated with liver fat content in women but not men [76], but we could not conduct subgroup analysis by sex in the current study as the lack of sex-specific data. Although the mechanism underlying the association between SHBG and liver fat regulation remains unclear, animal experiments implied that increased SHBG level can downregulate the expression of the crucial enzymes involved in the hepatic lipogenesis, such as the adenosine triphosphate (ATP) citrate lyase (production of precursor for fatty acid), Acetyl-CoA-carboxylase and fatty acid synthase (further restriction of fatty acid synthesis) in the liver [78, 79], which could consequently reduce liver fat content. In addition, in vitro experiments showed that SHBG can repress inflammatory cytokines, including interleukin-6 and tumor necrosis factor-alpha in adipocytes and macrophages, modulating inflammatory processes [79].

The main strength of our study is that it allowed for assessment of the causal role of biomarkers using MR, an approach that is robust to unmeasured error due to confounding. Colocalization analysis has been proven a powerful tool in revealing the pleiotropic effects of certain loci on multiple traits. The large sample sizes of both the GWAS ensure adequate statistical power to show clinically relevant associations between protein levels and risk of visceral and ectopic fat. In addition, we confined our analysis to individuals of European ancestry, which minimized the population stratification bias.

Some limitations of our analysis are worth noting. Firstly, our approach tested effects of circulating protein levels and could not capture potential tissue-specific effects. Secondly, the causal associations should be interpreted with caution, as several assumptions of the methods are untestable, and potential sample overlap, heterogeneity of the IVs and residual horizontal pleiotropy might bias the results. Finally, our findings were based on GWAS of European ancestry and cannot be generalized to other ancestries. This underscores the importance of large-scale biobanks including individuals of non-European ancestry.

In conclusion, our results provided evidence that genetically altered levels of ten circulating proteins were associated with the risk of visceral and ectopic fat, which might be the potential drug targets for future therapies. Future studies are needed to validate these candidate proteins in independent longitudinal case-control cohorts and non-European ancestry populations.

### Electronic supplementary material

Below is the link to the electronic supplementary material.

Supplementary Material 1

## Data Availability

No datasets were generated or analysed during the current study.

## Abbreviations

BMI: Body mass index
DBP: Diastolic blood pressure
GWAS: Genome-wide association study
HDL-C: High-density lipoprotein cholesterol
HOMA-B: Homeostasis model assessment of beta-cell function
HOMA-IR: Homeostasis model assessment of insulin resistance
IV: Instrumental variable
IVW: Inverse-variance weighting
LDL-C: Low-density lipoprotein cholesterol
MR: Mendelian randomization
NAFLD: Nonalcoholic fatty liver disease
SAT: Subcutaneous adipose tissue
SBP: Systolic blood pressure
SHBG: Sex hormone binding globulin
TC: Total cholesterol
TG: Triglycerides
WHR: Waist-to-hip ratio
VAT: Visceral adipose tissue
